# Intraoperative Ultrasound in the Management of Rare Lesions Involving the Intradural Extramedullary Spinal Compartment: A Quick, but Effective Helping Hand to Define the Optimal Surgical Strategy

**DOI:** 10.3390/cancers17223607

**Published:** 2025-11-08

**Authors:** Alessandro Pesce, Luca Di Carlo, Mauro Palmieri, Federica Novegno, Andrea Iaquinandi, Luca Denaro, Daniele Armocida, Antonio Santoro, Maurizio Salvati, Tamara Ius, Alessandro Frati

**Affiliations:** 1 Systems Medicine Department, University of Rome Tor Vergata, 00133 Rome, Italy; ale_pesce83@yahoo.it (A.P.); andrea.iaquinandi1987@gmail.com (A.I.);; 2 Human Neuroscience, University of Rome Sapienza, 00185 Rome, Italy; 3 Academic Neurosurgery, Department of Neurosciences, University of Padua, 35121 Padua, Italy; 4 Neuroscience Department, University of Turin, 10124 Turin, Italy

**Keywords:** intraoperative ultrasound (IOUS), intradural extramedullary tumors, hemangioblastoma, arachnoid cyst, neurenteric cyst, spinal chronic subdural hematoma, tethered cord syndrome, shear wave elastography

## Abstract

Intraoperative ultrasound (IOUS) is an increasingly adopted adjunctive intraoperative visualization method in spinal tumor surgery, offering real-time imaging that improves lesion localization, exposure planning, and resection control. This paper focuses on IOUS findings in rare intradural entities (neuroenteric/respiratory cysts, chronic spinal subdural hematoma, tethered cord/scarring, intradural extramedullary hemangioblastomas, and arachnoid cysts) where evidence remains limited. Across these lesions, IOUS typically depicts cysts as anechoic or hypoechoic cavities with definable walls and occasional septations; CSSDH is also delimited by hypoechoic subdural collections bounded by echogenic membranes; hemangioblastomas, as well as circumscribed, homogeneous nodules often with cystic components; and arachnoid webs/cysts with their boundaries and subtle subarachnoid communications. Doppler and micro-Doppler can delineate feeding and draining vessels in highly vascular tumors, while shear wave elastography provides quantitative stiffness changes that support effective detethering. IOUS complements preoperative MRI, shortens exposure, helps tailor bone and dural openings, and allows immediate assessment of residual disease. Taken together, current data and our experience support IOUS as a safe, cost-effective, and versatile intraoperative tool for rare intradural spinal pathology, while underscoring the need for prospective studies to refine sonographic criteria and validate outcome benefits.

## Simple Summary

Rare lesions inside the coverings of the spinal cord are hard to spot and remove safely. Scans performed before surgery do not always show exact edges or nearby vessels, so surgeons must decide how wide to open the bone and the dura, and when removal is complete. We evaluate intraoperative ultrasound as a real-time guide at the operating table. We describe how these lesions appear on ultrasound, how blood flow can be mapped during surgery, and how changes in tissue stiffness can confirm release in the tethered cord. Our goal is to give clear, practical cues that help surgeons find the lesion, plan the approach, and check for residual disease while limiting manipulation of the cord. Sharing these patterns may speed adoption, improve safety, and create a common language for future studies.

## 1. Introduction

**Intraoperative ultrasound (IOUS)** has become an increasingly important adjunct in spinal tumor surgery, offering real-time imaging that improves the precision and safety of surgical procedures. By enabling visualization of the lesion and surrounding spinal cord before durotomy, IOUS allows surgeons to optimize dural opening and surgical exposure, minimizing manipulation of healthy tissue and enhancing operative planning [1].

IOUS is particularly valuable in the resection of both **intramedullary tumors** (e.g., ependymomas, astrocytomas) and **extramedullary tumors** (e.g., meningiomas, schwannomas). High-frequency probes (10–12 MHz) provide detailed resolution, enabling accurate delineation of tumor margins [2,3,4]. For highly vascular lesions, such as hemangioblastomas or dural arteriovenous fistulas, the integration of Doppler ultrasound improves intraoperative assessment of vascular anatomy and assists in safer dissection [5,6].

When compared to intraoperative MRI (iMRI) and intraoperative CT (iCT), IOUS offers practical advantages: it is more cost-effective, requires minimal additional operative time, and does not expose patients or staff to ionizing radiation [7,8]. Moreover, its learning curve is relatively short, and studies indicate that IOUS influences intraoperative decision-making in spinal surgery in over 60% of cases [9].

One of its most significant contributions lies in the evaluation of the **extent of resection**. IOUS has demonstrated a strong correlation with postoperative MRI in identifying residual tumor, supporting its role in maximizing gross total resection (GTR) while minimizing unnecessary resection of normal tissue to limit postoperative neurological deficits to a minimum [10]. Furthermore, case series emphasize their value in limiting surgical exposure and bone removal, without compromising oncological outcomes [2].

Current evidence supports the integration of IOUS into spinal oncology practice as a cost-effective imaging modality that **enhances lesion localization and assessment of resection**. Evidence for some statements is extrapolated from intracranial literature [5,8] and from direct surgical experience. This work addresses an underreported area by **describing IOUS features in rare intradural lesions**, an area where the literature remains limited and where intraoperative imaging can provide critical insights for surgical decision making (Table 1). The selection of these specific rare intradural extramedullary lesions, including neuroenteric and respiratory cysts, chronic spinal subdural hematoma, tethered cord with scarring, extramedullary hemangioblastomas, and arachnoid cysts, is based on their shared location within the intradural space and the common surgical challenges they pose. These entities often present with ambiguous preoperative imaging, making intraoperative differentiation difficult, and there is minimal literature documenting their echo characteristics on intraoperative ultrasound. By grouping them, this review provides a focused description of their sonographic patterns to assist surgeons in unclear scenarios, such as optimizing dural opening or confirming resection. This approach addresses a notable gap, as opposed to common lesions where such guidance is already abundant.

Intradural extramedullary spinal lesions encompass a wide range of pathologies, from common entities such as schwannomas and meningiomas to rare ones like neuroenteric cysts and extramedullary hemangioblastomas. For common lesions, intraoperative ultrasound is already well established, typically depicting them as hyperechoic, well-circumscribed masses with distinct interfaces to surrounding neural structures, facilitating precise localization and resection control without the need for further comprehensive reviews. In contrast, rare lesions present unique challenges due to limited documentation of their sonographic patterns in the literature. This narrative review focuses on these rare entities to address this gap, providing detailed descriptions of their echo characteristics based on institutional experience. By contrasting with common lesions, this work offers a broader overview of intraoperative ultrasound’s role, guiding surgeons in ambiguous intraoperative scenarios where preoperative imaging may not fully clarify lesion boundaries or relationships. Intraoperative ultrasound is performed using high-frequency linear probes (10 to 12 MHz) to achieve optimal resolution for spinal structures. The standard protocol involves saline immersion as an acoustic coupling medium after laminectomy but before durotomy, allowing for clear visualization through the intact dura. Scanning is conducted systematically in sagittal and axial planes to localize the lesion, assess its margins, and evaluate relationships with the spinal cord. For vascular lesions, Doppler or micro-Doppler modes are integrated to map feeding and draining vessels. In cases of tethering, shear wave elastography provides quantitative stiffness measurements pre- and post-detethering. This setup, based on our institutional experience, ensures reproducibility and enhances practical utility for surgeons.

## 2. Disembryogenetic Cysts

**Neuroenteric** (often termed neurenteric) and **respiratory intradural cysts** constitute exceptionally rare congenital lesions arising from developmental missteps during embryogenesis, typically due to failure of separation between endoderm and neuroectoderm during the third week of development. These cysts are lined by epithelium resembling gastrointestinal or respiratory mucosa, and they most frequently manifest as intradural extramedullary masses in the lower cervical or upper thoracic spinal cord, accounting for just 0.7–1.3% of spinal lesions. Given their rarity, preoperative radiological diagnosis is challenging, as they may mimic more common cystic entities such as arachnoid cysts or cystic neoplasms on MRI [11,12,13].

**Figure 1:**
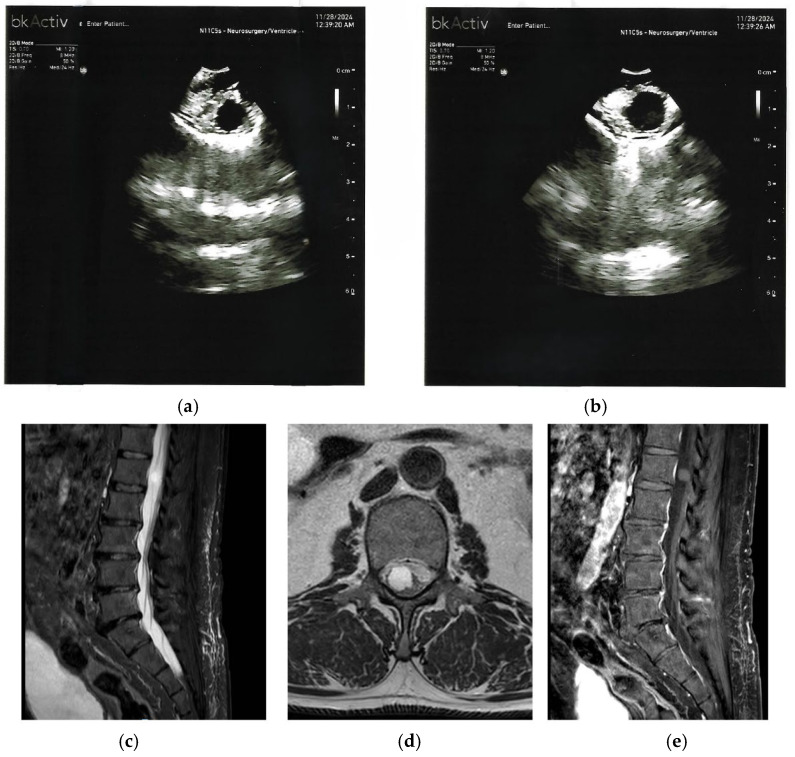
(**a**,**b**) Pre-durotomy intraoperative ultrasound shows a thin-walled, anechoic cyst (arrow in (**a**) indicates fine internal septation), producing mass effect with eccentric displacement of the spinal cord (labeled ‘cord displacement’ in (**b**)). The anechoic cavity on IOUS (**a**,**b**) corresponds to the CSF-like signal on MRI (**c**,**d**). (**c**) Sagittal T2-weighted MRI demonstrates a well-circumscribed intradural, extramedullary cystic lesion with CSF-like signal and focal cord compression (arrow indicates compression). (**d**) Axial T2-weighted MRI at the level of maximal compression confirms an intradural cyst eccentrically displacing the cord (labeled ‘cord displacement’). (**e**) Sagittal post-contrast T1-weighted MRI shows no enhancing solid component.

Because the literature on IOUS in these cyst types is minimal, this work contributes new insight. Generally, IOUS in intradural spinal cystic lesions, such as arachnoid or epidermoid cysts, enables precise localization before durotomy, tailored myelotomy planning, and visual confirmation of the cyst cavity and any septations or residual contents [14,15]. Based on our experience with similar cystic lesions and extrapolation from analogous intradural entities (e.g., arachnoid cysts [14,15]), IOUS typically reveals an anechoic or hypoechoic cavity with well-defined margins (Figure 1), often with an echogenic lining corresponding to the cyst wall; septations or internal echoes may indicate proteinaceous or mucinous content. Limited reports on analogous cystic lesions suggest these imaging characteristics distinguish them from solid intramedullary tumors, which tend to appear hyperechoic and heterogeneous on IOUS [16].

Moreover, IOUS provides essential guidance for surgical exposure by identifying lesions under intact dura. This allows for the determination of the extent and location of laminectomy or laminoplasty, which minimizes bone removal and facilitates an accurate dural opening [14,17]. During cyst resection, IOUS can verify complete evacuation of the cyst contents and integrity of the spinal cord architecture post-resection. Additionally, IOUS has been shown to accurately depict perilesional or intralesional cysts in analogous cases of intramedullary lesions with cystic components, such as ependymomas or hemangioblastomas, aiding in the planning and verification of safe resection [15,16].

In summary, although IOUS findings in neuroenteric and respiratory intradural cysts are not well documented, extrapolation from similar cystic and intradural entities suggests that IOUS would provide excellent visualization of the margins of the lesion, its internal architecture, and its relation to neural structures during surgery. This would support surgical precision and safety. This study thus fills a significant gap by characterizing IOUS features that are specific to these rare cysts.

## 3. Spinal Chronic Subdural Hematoma (CSSDH)

**Spinal chronic subdural hematomas (CSSDH)** are exceedingly rare collections of blood that accumulate in the subdural space of the spinal canal, typically developing over weeks following minor trauma, anticoagulant use, or spontaneously [18,19]. They often produce progressive symptoms such as back pain, lower limb weakness, and bowel or bladder dysfunction. They account for only a fraction of spinal space-occupying lesions and often have a better prognosis when identified and treated promptly [20]. MRI is the diagnostic gold standard, with CSSDH often appearing **hyperintense** on both T1- and T2-weighted sequences during the late subacute to chronic phase, due to methemoglobin or early hemosiderin deposition; late chronic lesions may appear **isointense** or **hypointense**, reflecting progression toward hemosiderin and fibrotic stages [21].

While there is scant literature specifically describing intraoperative ultrasound (IOUS) appearances of CSSDH, extrapolation from analogous pathologies and IOUS has a significant practical utility. During surgery, IOUS can be employed after bony removal and dura exposure, using saline coupling to visualize the dura, any subdural or intradural fluid collections, and underlying neural structures [22]. Based on our surgical experience and extrapolation from reported drainage cases [20], CSSDH typically appears as a hypoechoic or anechoic collection beneath the echogenic dura, often with internal echoes or septations if partially liquefied or encapsulated, and surrounded by a consistently echogenic membrane corresponding to chronic fibrous tissue. The hematoma’s liquid nature in late subacute stages (as seen on MRI and confirmed by “motor-oil”-like aspirate in reported drainage cases) may facilitate ultrasound visualization of the cavity boundaries and their relationship to the spinal cord and nerve roots [23].

**Figure 2:**
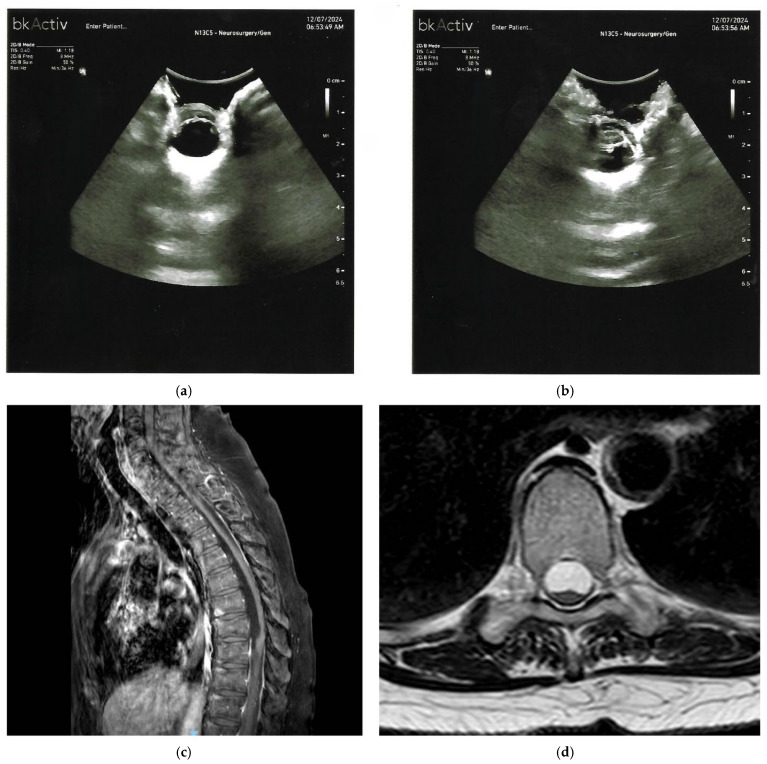
(**a**,**b**) Pre-durotomy intraoperative ultrasound demonstrates a predominantly anechoic/hypoechoic subdural collection immediately beneath the echogenic dura (arrow in (**a**) indicates echogenic membrane), bounded by a continuous echogenic membrane and containing fine internal echoes/septations (labeled ‘septations’ in (**b**)), consistent with a chronic hematoma and explaining the cord compression. The hypoechoic collection on IOUS (**a**,**b**) correlates with the elongated dorsal subdural collection on MRI (**c**). (**c**) Sagittal MRI shows an elongated dorsal subdural collection along the thoracic canal with mass effect and ventral displacement of the spinal cord (arrow indicates displacement). (**d**) Post-evacuation axial T2-weighted MRI showing re-expansion of the cord with restoration of the dorsal CSF space and near-complete resolution of the posterior subdural collection.

IOUS also helps in tailoring the extent of laminectomy and dural opening precisely over the hematoma, thus minimizing unnecessary bone removal and exposure. Limited reports indicate that real-time assessment using IOUS facilitates confirmation of complete evacuation or adequate drainage of the subdural collection, which is especially relevant when minimally invasive drainage is attempted in select chronic or liquefied CSSDH cases [23]. Furthermore, in scenarios involving multiple or extensive CSSDH, IOUS could guide exploration of additional subdural collections that might not be evident on preoperative imaging [24]. **IOUS can demonstrate, in CSSDH, effective decompression with restoration of spinal cord caliber and midline position following evacuation. Practically, after a successful surgery, the upward displaced spinal cord returns to its natural position grossly at the center of the spinal canal **(Figure 2).

In summary, appear as **well-defined, hypoechoic subdural collections, bounded by echogenic membranes**. IOUS has the potential to guide surgical exposure and confirm evacuation in the management of CSSDH, ultimately enhancing operative safety and precision.

## 4. Spinal Cord Tethering

**Spinal cord tethering** refers to a pathologic fixation of the spinal cord within the spinal canal, commonly due to congenital anomalies, thickened filum terminale, lipomas, or, in some cases, postoperative scar adhesions as a result of previous intradural surgical procedures. In tethered cord syndrome (TCS), the cord is abnormally anchored, leading to traction, impaired neural perfusion, and progressive neurological deficits such as lower limb weakness, sensory loss, and bladder dysfunction [25]. Post-surgical scarring can exacerbate tethering, increasing the risk of retethering and clinical deterioration [26,27].

**Intraoperative ultrasound**, particularly B-mode and shear wave elastography (SWE), offers real-time imaging of the tethered spinal cord and surrounding scar tissue. In a recent human case series, IOUS-SWE was used intraoperatively to measure spinal cord stiffness before and after untethering. Stiffness decreased significantly, dropping from a median of around 93.84 kPa to approximately 9.35 kPa, which indicates the successful release of pathological tension [28]. This quantitative assessment provides an objective, reproducible marker for successful untethering. This is especially valuable when it is challenging to visually differentiate between scar tissue, the filum terminale, and neural tissue.

Beyond SWE, standard B-mode IOUS has facilitated delineation of anatomical relationships in occult spinal dysraphisms, which are common causes of tethering, principally in pediatric series. In a cohort of children with various lesions (including filum lipomas, diastematomyelia, and dorsal myeloschisis), IOUS reliably identified **cystic components** as **anechoic**, **lipomas** as **hyperechoic**, and could visualize neural structures, adhesion bands, and spurs in real time, aiding surgical planning and minimizing dissection risk [29]. Differentiating between chronic spinal subdural hematoma, tethering, and arachnoid cysts on intraoperative ultrasound can be challenging due to overlapping appearances, such as hypoechoic collections or effaced subarachnoid spaces that may mimic one another. For instance, chronic spinal subdural hematoma often shows internal echoes or septations within a hypoechoic subdural space, while tethering presents with hyperechoic adhesions and reduced cord excursion, quantifiable via stiffness changes on shear wave elastography. Arachnoid cysts typically appear as anechoic cavities with subtle boundaries. Clinical context, preoperative imaging correlation, and multimodal tools like Doppler for vascularity or elastography for tissue properties are essential for accurate intraoperative differentiation, reducing diagnostic ambiguity in these rare scenarios.

**Figure 3:**
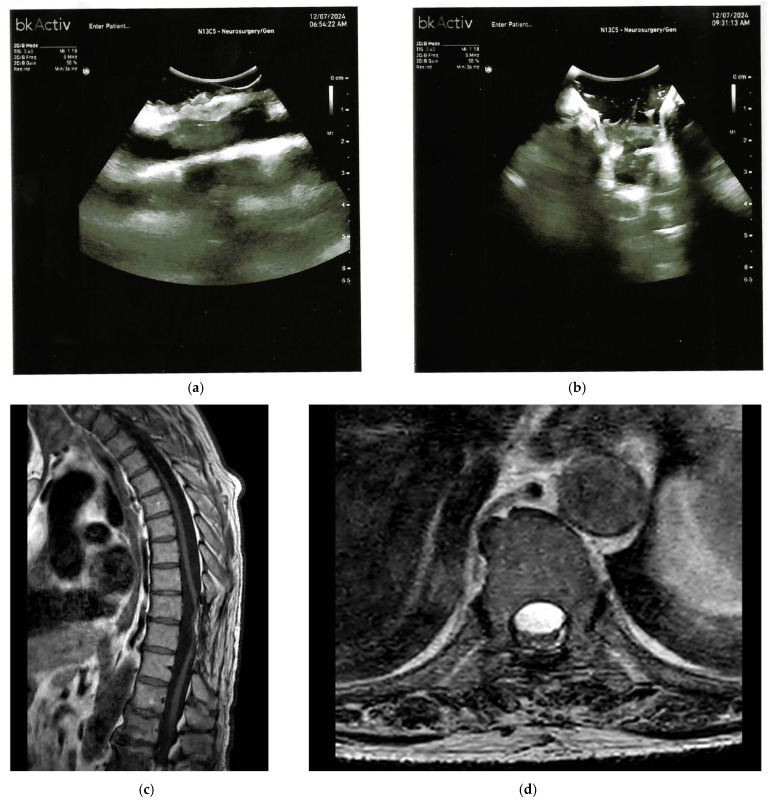
(**a**,**b**) Intraoperative B-mode ultrasound before release shows hyperechoic adhesions bridging dorsal dura and cord (arrow in (**a**) indicates adhesions) with near-complete effacement of the dorsal subarachnoid space and reduced cord excursion (labeled ‘effaced space’ in (**b**)). The hyperechoic adhesions on IOUS (**a**,**b**) correspond to the focal dorsal CSF loculation and anterior displacement on MRI (**c**,**d**). (**c**) Sagittal T2-weighted MRI demonstrates focal dorsal CSF loculation with anterior displacement of the thoracic cord (“scalpel sign”; arrow indicates displacement). (**d**) Axial T2-weighted MRI shows a crescentic dorsal subarachnoid collection compressing and anteriorly displacing the cord without a solid intradural mass findings consistent with tethering due to an arachnoid band/web (labeled ‘arachnoid band’).

Moreover, IOUS informs the surgeon’s intraoperative decision-making by confirming the anatomical plane between scar tissue and neural elements, guiding tailored dural and bone opening. It provides real-time feedback during dissection and helps verify adequate detethering without additional retraction or extended exposure (Figure 3). This is especially critical in previously operated cases or in cases of extensive fibrosis, where neurophysiologic monitoring alone cannot localize residual adhesions [29,30]. Intraoperative appearances of CSSDH, tethering, and arachnoid cysts may overlap on IOUS, complicating intraoperative differentiation, making the IOUS differential diagnosis difficult [29,30].

IOUS, also including elastography, provides both **qualitative** (echogenic differentiation of scar, lipoma, CSF spaces) and **quantitative** (tissue stiffness) insights during tethering surgery. It enhances the accuracy, safety, and confirmation of effective detethering during surgery, particularly in complicated scenarios involving dense scarring, anatomical ambiguity, and re-operative patients. As a complement to standard neurophysiologic monitoring, IOUS should become an invaluable tool in managing spinal cord tethering and associated scarring.

The quantitative results from shear wave elastography in tethered cord, showing a stiffness reduction from a median of 93.84 kPa to 9.35 kPa post-detethering, are derived from a recent human case series with a very small sample size, limited to only a few patients due to the rarity of these conditions. This novel application is promising for objective confirmation of release but must be interpreted cautiously, as potential technical confounders such as probe placement variability, tissue heterogeneity, or intraoperative artifacts could influence measurements. These preliminary findings highlight the need for larger prospective studies to validate reliability, establish normative ranges, and address these limitations in broader clinical contexts.

## 5. Hemangioblastomas

**Intradural extramedullary hemangioblastomas** are rare, benign, and highly vascular spinal tumors, typically associated with Von Hippel–Lindau (VHL) disease, yet occasionally arising sporadically. Unlike the more common intramedullary hemangioblastomas, these extramedullary variants are located within the dura but outside the spinal cord parenchyma, comprising approximately 21% of spinal hemangioblastomas (with 60% intramedullary, ~11% mixed intra- and extramedullary, and ~8% extradural) [31,32].

In line with the established use of intraoperative ultrasound in spinal tumor surgery, **IOUS** provides real-time imaging that supports lesion localization, approach planning, and assessment of the extent of resection [1]. Clinically, they often present with radicular pain or myelopathy, and surgical gross total resection is the treatment of choice, typically under neurophysiological monitoring to preserve function [31,32].

Due to their distinctive vascular architecture and frequent association with cystic components or syrinxes, IOUS provides critical real-time insight during surgery. In a pooled series of intradural tumors, hemangioblastomas (particularly intramedullary ones) consistently appeared as isoechoic, homogeneous nodules accompanied by cystic components. This distinguishes them from other lesions, such as ependymomas or meningiomas [17]. While this study is mainly focused on intramedullary lesions, the findings likely translate to extramedullary hemangioblastomas, where the lesion usually forms a well-circumscribed mass exterior to the cord. Based on a pooled series of analogous intradural tumors and our experience, IOUS typically visualizes a well-defined, homogeneous, and vascular nodule, often with an adjacent cystic cavity, facilitating precise localization and extent determination before durotomy.

Moreover, **real-time vascular imaging techniques** such as micro-Doppler ultrasound (µDoppler) have been recently implemented in spinal hemangioblastoma surgery. Limited reports on micro-Doppler in spinal hemangioblastoma surgery suggest these techniques visualize intraoperative vascular architecture, including feeding arteries and draining veins, in exquisite detail without the need for contrast agents, offering surgeons invaluable guidance in controlling bleeding and planning safe resection, particularly in deeply seated or highly vascularized tumors [33]. Given the rarity of intradural extramedullary hemangioblastomas, the intraoperative ultrasound appearance is extrapolated from intramedullary variants, typically presenting as well-circumscribed, homogeneous nodules often with cystic components. This similarity aids in guiding surgical strategies, though direct evidence remains limited.

Therefore, IOUS provides, in the context of surgical management of intradural extramedullary hemangioblastomas, precise **anatomical mapping** of the nodule and associated cysts, and **vascular profiling** with real-time visualization of feeding and draining vessels and their flow. Together, these capabilities enhance surgical precision, optimize exposure, reduce morbidity, and support complete tumor removal. Given the rarity of these lesions, future dedicated studies will further elucidate IOUS signature patterns specific to extramedullary hemangioblastomas and refine sonographic criteria to aid intraoperative decision-making [15,33,34].

## 6. Intradural Spinal Arachnoid Cysts

**Intradural spinal arachnoid cysts** are uncommon, benign lesions located within the dura mater but outside the spinal cord parenchyma. They are typically filled with cerebrospinal fluid (CSF) and can be congenital or acquired, often resulting from trauma, inflammation, or previous surgical interventions. Clinically, these cysts may present with symptoms ranging from asymptomatic to progressive myelopathy, including pain, weakness, and sensory disturbances.

Intraoperative ultrasound has emerged as a valuable tool in the management of these cysts. It provides real-time imaging, allowing for precise localization and assessment of the cyst’s relationship with surrounding neural structures. This capability is particularly beneficial during surgical interventions, where accurate identification of the cyst’s boundaries is crucial to minimize neural damage and ensure effective decompression [35].

Classification and management have been detailed by Klekamp [36]. Kalsi et al. [35] highlighted the utility of IOUS for assessing CSF flow and cyst/syrinx size; shunting is advised when excision is not feasible and compression or CSF flow restriction persists [36]. The authors emphasized that cyst shunting should be performed when the cyst cannot be excised and continues to compress the cord or restrict CSF flow [36].

Additionally, intraoperative ultrasound assists in visualizing the arachnoid web, a rare variant of spinal arachnoid cysts, where one or multiple focal membranes of arachnoid tissue obstruct the subarachnoid space. This visualization aids in ensuring complete resection of the arachnoid web and restoration of normal CSF flow [37,38].

Furthermore, intraoperative ultrasound can detect subtle communications between the cyst and the subarachnoid space, which may not be evident on preoperative imaging. Identifying these communications is essential for planning appropriate surgical strategies, such as fenestration or shunting, to prevent recurrence [39].

In conclusion, intraoperative ultrasound offers significant advantages in the surgical management of intradural spinal arachnoid cysts. Its real-time imaging capabilities enhance surgical precision, facilitate comprehensive resection, and contribute to improved patient outcomes. As such, it should be considered an integral component of the surgical toolkit for these challenging lesions. All major features of the lesions described are summarized in Table 2.

**Table 2:** Summary of MRI and IOUS characteristics of rare intradural extramedullary spinal lesions.

Lesion Type	MRI Characteristics	IOUS Characteristics
Neuroenteric or Respiratory Cysts	Well circumscribed, cerebrospinal fluid-like signal on T2; no enhancing solid component; focal cord compression.	Anechoic or hypoechoic cavity with well-defined margins, echogenic lining, occasional septations; eccentric cord displacement.
Spinal Chronic Subdural Hematoma (CSSDH)	Hyperintense on T1 or T2 (late subacute); isointense or hypointense (chronic); elongated dorsal collection with cord displacement.	Hypoechoic or anechoic subdural collection beneath echogenic dura; internal echoes or septations; bounded by echogenic membrane.
Spinal Cord Tethering or Scarring	Focal dorsal cerebrospinal fluid loculation; anterior cord displacement (“scalpel sign”); adhesions or thickened filum.	Hyperechoic adhesions bridging dura and cord; effaced subarachnoid space; reduced cord excursion; stiffness reduction post detethering (for example, 93.84 kilopascals to 9.35 kilopascals via shear wave elastography).
Intradural Extramedullary Hemangioblastomas	Well circumscribed nodule with cystic components or syrinx; enhancing on contrast; associated with von Hippel Lindau disease.	Well defined, homogeneous, isoechoic nodule; cystic components; vascular profiling via Doppler or micro-Doppler for feeders or drainers.
Intradural Spinal Arachnoid Cysts or Webs	Cerebrospinal fluid filled; subtle subarachnoid communications; cord compression if symptomatic.	Anechoic cavities with definable walls; subtle communications; boundaries and cerebrospinal fluid flow assessment.

## 7. Summary and Key Message

**Intraoperative ultrasound (IOUS)** has emerged as a versatile and increasingly used tool in the surgical management of rare intradural spinal lesions, encompassing neuroenteric and respiratory cysts, chronic subdural hematomas, spinal cord tethering and scarring, intradural extramedullary hemangioblastomas, and arachnoid cysts. These rare lesions, although distinct in pathogenesis and morphology, share the common challenge of precise localization and safe resection within the confined spinal canal.

Across all the lesions involving the intradural compartment, IOUS consistently provides real-time, high-resolution imaging that complements preoperative MRI, enhances surgical precision, minimizes bone and dural exposure, and enables immediate intraoperative assessment of resection completeness. Its cost-effectiveness, safety, and relative ease of integration into standard spinal surgery workflows make it particularly valuable for rare intradural lesions, where preoperative imaging may be insufficient to fully define complex anatomy or cystic and vascular components. A key limitation of this narrative review is the frequent extrapolation of intraoperative ultrasound findings from more common lesions or analogous pathologies, such as from intramedullary to extramedullary hemangioblastomas. While these descriptions are grounded in direct surgical experience and limited available reports, they introduce potential variability, as the actual imaging characteristics for these specific rare intradural extramedullary entities remain largely unverified in prospective studies. This underscores the need for future validation through larger cohorts to refine sonographic criteria and confirm their reliability in clinical practice.

Collectively, these findings underscore the unique role of IOUS as a dynamic intraoperative adjunct that improves surgical planning, execution, and outcomes in rare intradural spinal pathology.

## 8. Conclusions

Intraoperative ultrasound is a real-time imaging modality that enhances the surgical management of rare intradural spinal lesions. By providing precise anatomical and vascular visualization, IOUS improves lesion localization, guides safe resection, and helps confirm completeness, with potential benefits for patient outcomes in these challenging pathologies.

## Figures and Tables

**Table 1:** Comparison of intraoperative imaging modalities for intradural extramedullary spinal lesions.

Parameter	Intraoperative Ultrasound (IOUS)	Intraoperative MRI (iMRI)	Intraoperative CT (iCT)
Cost	Low (cost-effective, no specialized room required)	High (requires dedicated operating room and equipment)	Moderate (less expensive than iMRI but requires scanner)
Examination Time	Minimal (real time, less than 5 min per scan)	Prolonged (10 to 30 min per scan, interrupts surgery)	Short (5 to 10 min, but may require repositioning)
Effectiveness for Lesion Localization	High (real-time visualization of cystic or hypoechoic structures, margins, and cord displacement)	Excellent (superior soft tissue resolution for rare lesions)	Good (bone and gross anatomy, limited for soft tissue)
Effectiveness for Resection Control	High (immediate assessment of residuals, Doppler for vascularity, elastography for stiffness)	Excellent (gold standard for residual tumor detection)	Moderate (limited for intradural details without contrast)
Radiation Exposure	None	None	Present (ionizing radiation)
Real Time Capabilities	Excellent (dynamic imaging during surgery)	Limited (static scans)	Limited (static scans)
Advantages for Rare Lesions	Versatile for underreported echo patterns (for example, anechoic cysts, hypoechoic collections); minimizes exposure	Detailed anatomy, but less practical for quick decisions	Useful for bony landmarks but insufficient for cystic or vascular rarities
Disadvantages	Operator dependent; lower resolution for deep structures	High cost and time; not real time	Radiation risk; poor soft tissue contrast
References	Based on [1,7,8] from the manuscript	Extrapolated from [7,8]	Extrapolated from [7,8]

## Abbreviations

CSF	cerebrospinal fluid
CSSDH	chronic spinal subdural hematoma
CT	computed tomography
GTR	gross total resection
iCT	intraoperative computed tomography
iMRI	intraoperative magnetic resonance imaging
IOUS	intraoperative ultrasound
MRI	magnetic resonance imaging
SWE	shear wave elastography
TCS	tethered cord syndrome
VHL	Von Hippel–Lindau (disease)
µDoppler	micro-Doppler ultrasound
